# Post-traumatic stress symptoms in mental healthcare workers during the COVID-19 outbreak

**DOI:** 10.4102/sajpsychiatry.v29i0.2098

**Published:** 2023-10-24

**Authors:** Allison K. Human, Nadira Vahed, Belinda Marais

**Affiliations:** 1 Department of Psychiatry, Faculty of Health Sciences, School of Clinical Medicine, University of the Witwatersrand, Johannesburg, South Africa

**Keywords:** post-traumatic stress symptoms, mental healthcare workers, COVID-19, coping skills, perceived social support, Gauteng province, South Africa

## Abstract

**Background:**

In the context of disease outbreaks, healthcare workers are exposed to multiple physical and psychological stressors, which may result in severe mental health outcomes. Although existing literature explores this impact, it is focused on frontline workers, with limited evidence exploring the mental well-being of mental healthcare workers (MHCWs).

**Aim:**

To explore post-traumatic stress symptoms (PTSS) and associated factors among MHCWs within the context of the coronavirus disease 2019 (COVID-19) pandemic.

**Setting:**

Four academic hospitals in the Gauteng province, South Africa, with specialised psychiatric units.

**Methods:**

A cross-sectional study design was used. Participants were selected using a simple random sampling technique and invited to participate in structured interviews. Measurement tools included a demographic questionnaire, the post-traumatic stress disorder checklist for DSM-5 and the Brief Resilient Coping Scale.

**Results:**

A total of 120 MHCWs participated. The prevalence of PTSS was 11.7%. The MHCWs’ profession was a significant predictor of the occurrence of PTSS (*p* = 0.046), with nurses being the most affected. Other socio-demographic, employment, COVID-19-related factors and coping skills were not predictors of PTSS.

**Conclusion:**

An elevated prevalence of PTSS has been found and was significantly associated with the profession of the MHCW. It is recommended that existing employee wellness programmes be strengthened to promote mental well-being and improve resilience among MHCWs, particularly vulnerable employee groups.

**Contribution:**

This study provides insight into the prevalence of PTSS among MHCWs following the COVID-19 outbreak, as well as associated factors.

## Introduction

On 11 March 2020, the World Health Organization declared the coronavirus disease 2019 (COVID-19) outbreak a global pandemic.^1^ As per the World Health Organization Africa region report, close to nine million cases had been reported in the African region, with South Africa accounting for four million cases in this region as of 08 August 2022.^2^ At that stage, healthcare worker (HCW) infections continued to rise, and South Africa remained one of the five countries accounting for approximately 70% of all infections reported in African HCWs.^2^

In addition to the evidenced increased risk of infection experienced during disease outbreaks, HCWs are exposed to several physical and psychological stressors, which may result in severe mental health outcomes.^3^

Following the severe acute respiratory syndrome (SARS) outbreak, HCWs reported elevated stress levels, as well as significant emotional distress and psychiatric morbidity.^4,5,6,7,8^ Long-term psychological morbidity in the form of elevated stress levels, anxiety, depression and post-traumatic stress disorder (PTSD)-like symptoms has been documented up to 2 years after the SARS outbreak.^4,7,8^

Similarly, following the Middle East respiratory syndrome outbreak, HCWs expressed moderate anxiety towards Middle East respiratory syndrome-coronavirus infection related to the novelty of the virus, its ability to cause severe disease or death and the lack of availability of specific treatments or vaccines.^9^ Additionally, higher levels of concern were associated with younger HCWs, being non-physician, direct patient contact and working within the central region of the outbreak.^10^

Recent literature produced during the COVID-19 outbreak has displayed significant negative psychological effects among HCWs.^11,12,13,14^ These effects occur in the face of an increasing workload, limited personal protective equipment, widespread media coverage and lack of specific treatment regimens.^13^ A meta-analysis of 12 studies, primarily performed in China, found the prevalence of anxiety, depression and insomnia among HCWs during this outbreak to be 23.3%, 22.8% and 38.9%, respectively.^15^ In a study completed among HCWs in Oman, the overall psychological well-being of these workers was poor. This was more evident among females, young HCWs and those who cared directly for COVID-19-positive patients.^16^

Trauma is defined as a ‘stressful occurrence that is outside the range of the usual human experience and that would be markedly distressing to almost anyone’. It involves a ‘perceived intense threat to life, physical integrity, intense fear, helplessness, or horror.^17^ Post-traumatic stress symptoms (PTSS) follow an acute traumatic occurrence and are characterised by a typical symptom pattern of intrusive thoughts, persistence of trauma, relevant stimuli avoidance, emotional numbing and physiological hyperarousal.^18^

Persistently experiencing PTSS may lead to the development of PTSD. Research has identified that PTSS that developed within days of trauma was a robust predictor for the development of PTSD.^19^ By means of its threat to human life and the associated emotions induced, the presence of a global pandemic in the form of COVID-19 meets the definition of a traumatic event.^18^

The PTSD checklist for DSM-5 (PCL-5) utilised in this study is one of the most widely used self-report measures of PTSD, demonstrating excellent reliability and validity. The tool quantifies and monitors symptoms over time, screening for PTSD and assisting in making a provisional diagnosis of PTSD. Although there is only one version of the PCL-5, there are three formats that differ in how Criterion A is assessed. The first version, used in this study, does not assess Criterion A, the second version defines Criterion A and provides examples of qualifying events, and the third version includes the Life Events Checklist for DSM-5 and a more detailed assessment of Criterion A. The PCL-5 is meant to assess patient symptoms in the past month. Versions that assess symptoms over a different time frame (the past day, past week, or past 3 months) have not been validated.^20,21^

Literature related to the SARS outbreak investigating PTSS in three subgroups, namely patients, HCWs and the general public, found the incidence of PTSS among HCWs to be 25.8%.^22^ In a prospective and follow-up study completed in Taiwan among 102 nurses working in both SARS and non-SARS units, no significant difference was found in the PTSD prevalence rate.^23^ This is in keeping with the findings of a Toronto-based study, which looked at the long-term psychological and occupational effects of providing healthcare during the SARS outbreak.^6^ This is suggestive that epidemic experiences could be considered traumatic for HCWs across departments. These emotions may result from uncertainty regarding redeployment to understaffed high-risk areas, an imbalance between skill set and work demand and limited psychological preparation.^17,24^

In a rapid review assessing the psychological impact of epidemic and pandemic outbreaks on HCWs, PTSS were examined in 23 studies. During outbreaks, the prevalence of PTSD-like symptoms ranged between 11% and 73.4%, with the prevalence range gradually decreasing from 10% to 40% 1–3 years after the outbreaks.^3^

Regarding risk and protective factors, various organisational, social, personal and psychological predictors have been associated with a higher prevalence of PTSS in HCWs.^25^ In terms of organisational predictors, occupational role, working in high-risk environments and being quarantined are associated with a higher prevalence of PTSS.^25,26,27^

Socially, organisational support and support from family or friends have proved to protect HCWs’ mental health outcomes.^23,24,27^ Personal characteristics that increase the risk of PTSS in HCWs include being single, female, a young healthcare professional with less work experience and earning a lower household income.^8,13,27,28^ Psychological predictors, such as maladaptive coping styles and having a negative emotional experience of the outbreak, are associated with an increased likelihood of PTSS among HCWs.^24,26,27^

Mental healthcare workers (MHCWs) play an important role amid a pandemic by providing psychological support and guidance. They are often considered role players in developing strategic protocols to alleviate distress and improve coping skills among their colleagues and the public.^29^

Although there is significant literature exploring the impact of epidemics on HCWs’ mental health, many of these are focused on direct frontline workers with minimal evidence surrounding the mental health of MHCWs.

## Aim and objectives

This study aimed to explore PTSS and associated factors among MHCWs in the Gauteng province within the context of the COVID-19 pandemic.

The study objectives were:

- To describe the socio-demographic and employment factors, the extent of COVID-19 exposure and the perceived social support of MHCWs
- To determine the prevalence of PTSS among MHCWs using the PCL-5
- To determine MHCWs’ coping skills using the Brief Resilient Coping Scale (BRCS).
- To determine whether the given factors are associated with PTSS in the form of risk factors and protective factors.

## Methods

### Study design and setting

This study was a cross-sectional hospital-based survey across four academic hospitals with specialised psychiatric units within the Gauteng province, South Africa namely: Tara The H. Moross Centre (TARA), Helen Joseph Hospital (HJH), Sterkfontein Hospital (SFH) and Chris Hani Baragwanath Academic Hospital (CHBAH).

### Study population and sampling strategy

The study population included MHCWs who formed part of the psychiatric multidisciplinary team. Participants were selected based on the following inclusion and exclusion criteria.

Inclusion criteria:

- Mental healthcare workers within the multidisciplinary team at the specified psychiatric units who were employed from at least the beginning of the COVID-19 outbreak in South Africa, that is, 05 March 2020, based on the first positive case identified by the National Institute of Communicable Disease;
- Multidisciplinary team members, including psychiatrists, psychologists, psychiatric nursing staff, occupational therapists and social workers within these units;
- Participants had to be over the age of 18.

Exclusion criteria:

- Non-medical staff employed at the specified psychiatric units who were not directly involved in patient management
- Participants with a previous diagnosis of PTSD prior to the COVID-19 pandemic;
- Participants who were not fluent in English, as the questionnaires were administered by an English-speaking investigator using measurement tools produced in English.

To determine the sample size, an assessment of Z-scores of expected frequencies for variables considered in the study indicated that statistical significance could be expected with a minimum sample of 120, assuming a large effect size. Participants were selected using a simple random sampling technique. Potential participants at each hospital site were approached by the principal investigator and invited to participate in the study. A questionnaire relating to the objectives of this study was administered via on-site interviews with each participant facilitated by the principal investigator, who provided an introductory overview of the questionnaire, as well as clarity of content where requested.

### Data collection

Data collection took place from July 2021 to June 2022 until the required sample size was obtained.

### Measurement tools

The PCL-5 was used to determine the prevalence of PTSS among the participants. The PCL-5 is a 20-item self-report measure that assesses the 20 DSM-5 symptoms of PTSD. Respondents were asked to rate the severity of PTSD symptoms using a five-point Likert scale ranging from 0 to 4. A total score is reached by adding the scores for each item. A PCL-5 cut-off score of between 31 and 33 indicates probable PTSD.^20^ For this study, a cut-off score equal to or greater than 31 was used. The PCL-5 has demonstrated strong reliability (84%) and convergent and discriminant validity.^20,21^

A demographics questionnaire and the BRCS were used to determine the risk and protective factors associated with the mental health outcomes within this study population. The demographics questionnaire, designed by the principal investigator, detailed the participant’s personal and social demographics, living conditions and an elaboration on access to healthcare, as well as the extent of exposure to COVID-19 during the outbreak. In addition, it measured their degree of perceived social support.

The BRCS is a four-item measure designed to capture an individual’s tendency to cope with stress in a highly adaptive manner. Each question is measured using a five-point Likert scale. Participants are then rated as low (score of 4 to 13), medium (score of 14 to 16) or high resilience copers (score of 17 to 20). This tool has displayed fair validity and reliability for a four-item scale.^30^

### Data analysis

Statistical analyses were conducted using R software (version 4.0.0). Data sets were assessed for departure from normality using the Shapiro–Wilk test. Model significance was set at 0.05, and all tests were two tailed. Data were categorical and represented as counts and percentages and plotted in graphs or presented in tables. For the prevalence of PTSS among the MHCWs, the number with PTSS (PCL-5 cut-off score equal to or greater than 31) versus those without PTSS was calculated as a percentage of the total sample size with a 95% confidence interval (CI). Data were analysed using Pearson’s chi-squared by association (2 × k matrices) or goodness of fit, followed by binary post hoc tests or Fisher’s exact tests, as appropriate. Fisher’s exact tests were used to analyse 2 × 2 matrices. The relationship between the MHCWs’ coping scores and PTSS was analysed using the Mann–Whitney U test.

### Ethical consideration

The research protocol was approved by the University of the Witwatersrand Human Research Ethics Committee. Site approval was obtained from all hospitals involved: M210114 (24/06/2021). Participation was voluntary, and participants were allowed to withdraw at any stage. The surveys did not contain overt identifying information. A signed informed consent was required prior to the initiation of the interview. Participants were informed that they could be provided with acute psychological support via the psychological services available at any of the institutions involved in this study should this be required.

## Results

### Study participants

A total of 120 MHCWs participated in the study, representing four regional hospitals: CHBAH (*n* = 30), HJH (*n* = 27), SFH (*n* = 30) and Tara Hospital (*n* = 33). There were 28 doctors, 37 nurses, 20 occupational therapists, 20 psychologists and 15 social workers.

### Socio-demographic and employment factors

The socio-demographic and employment factors of the participants are presented in Table 1 and Table 2. This includes factors such as age, sex, relationship status, access to healthcare as well as participant’s position of employment and degree of work experience.

**TABLE 1:** Socio-demographic characteristics of mental healthcare workers from four Gauteng hospitals.

Variable	*n*	%
**Age (years)**		
20–29	47	39
30–39	45	38
40–49	17	14
50–59	11	9
**Sex**		
Female	101	84
Male	19	16
**Relationship status**		
In a relationship	42	35
Married	37	31
Single	37	31
Divorced	3	3
Widowed	1	1
**Number of dependents**		
0	68	57
1–2	32	27
3–4	15	13
≥ 5	5	4
**Religion**		
Christian	88	73
Muslim	8	7
Areligious	7	6
Hindu	7	6
Jewish	2	2
Other	8	7
**Number of people in household**		
1–5	108	89
> 5	12	10
**Type of housing**		
Formal	118	98
Informal	2	2
**Access to amenities**		
Yes	120	100
No	0	0
**Access to healthcare**		
Private	93	78
Public	27	23

**TABLE 2:** Employment factors of mental healthcare workers from four Gauteng hospitals.

Variable	*n*	%
**Position of employment**		
Nurse	37	31
Doctor	28	23
Occupational therapist	20	17
Psychologist	20	17
Social worker	15	13
**Years of work experience**		
0–5	65	54
5–10	28	23
> 10	27	23

### Extent of COVID-19 exposure and perceived social support

More respondents cared for a COVID-19-positive patient and had someone close infected with COVID-19. Most respondents strongly agreed that they had perceived social support (Table 3).

**TABLE 3:** COVID-19-related characteristics of mental healthcare workers from four Gauteng hospitals.

Variable	*n*	%
**Care of COVID-19 patients**		
Yes	31	26
No	89	74
**Someone close infected with COVID-19**		
No	8	7
Yes	112	93
**Demise of someone close because of COVID-19**		
No	63	53
Yes	57	48
**Infection of participant with COVID-19**		
No	65	54
Yes	55	46
**Need for participant to self-isolate**		
No	45	38
Yes	75	63
**Participant’s perceived level of social support**		
Strongly agree	93	78
Agree	26	22
Disagree	1	1

COVID-19, coronavirus disease 2019.

### Prevalence of post-traumatic stress symptoms according to the PCL-5

Fourteen MHCWs were classified as having PTSS based on their PCL-5 scores; thus, the prevalence of PTSS among the MHCWs was 11.7% (95% CI: 0.65, 0.19). Participants with PTSS had a mean score of 45.5 using the PCL-5. In the context of a cut-off score greater than or equal to 31, this score relates to participants having probable PTSD. In relation to the position of employment, 22% of nurses (*n* = 8) reported PTSS. Among occupational therapists, 15% (*n* = 3) reported PTSS; similarly, 15% of the psychologists (*n* = 3) reported PTSS. None of the doctors or social workers in the study sample reported PTSS.

### Coping skills of mental healthcare workers according to the Brief Resilient Coping Scale

According to the 120 MHCWs’ BRCS, 24 (20%) were classified as low-resilience copers, 54 (45%) were classified as medium-resilience copers, and 42 (35%) were classified as high-resilience copers. Of these participants, two who were classified as low-resilience copers had PTSS, 10 who were classified as medium-resilience copers had PTSS and two who were classified as high-resilience copers had PTSS. Mental healthcare workers with PTSS had lower BRCS scores (i.e. poorer coping skills) than those with no PTSS: mean (standard deviation [s.d.]) = 14.36 (2.10) versus 15.55 (2.61) (Welch *t*-test *t* = −1.94, *df* = 18, *p* = 0.069).

### Factors associated with post-traumatic stress symptoms

Regarding predictors of PTSS, the MHCW’s profession was a significant predictor (χ^2^ = 9.67, *df* = 4, *p* = 0.046), with the proportion of nurses with PTSS (22%) being significantly greater than in other MHCWs.

**FIGURE 1:**
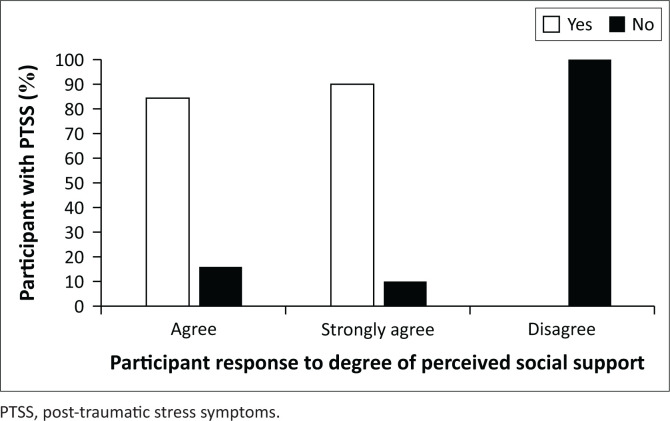
Percentage of mental healthcare workers from four Gauteng hospitals that suffered post-traumatic stress symptoms by the level of perceived social support.

In addition, poor levels of perceived social support were significantly associated with PTSS (χ^2^ = 8.28, *df* = 2, *p* = 0.015). A greater proportion of MHCWs with PTSS disagreed (100%, *n* = 1) with having perceived social support versus those who agreed (15%, *n* = 26) and those who strongly agreed with having perceived social support (10%; *n* = 93) (Figure 1). However, the sample size of those who disagreed with having perceived social support was too small to make meaningful conclusions.

None of the other socio-demographic, employment or COVID-19-related factors were significant predictors of the occurrence of PTSS (Table 4 and Table 5).

**TABLE 4:** Relationship between socio-demographic factors and the occurrence of post-traumatic stress symptoms among mental healthcare workers from four Gauteng hospitals.

Variable	PTSS count (%)				Statistics			
	No		Yes		*χ* ^2^	*df*	*p*-value	Fisher’s *p*-value
	*n*	%	*n*	%				
**Hospital**	-	-	-	-	5.68	3	0.129	-
CHBAH	26	87	4	13	-	-	-	-
HJH	27	100	0	0	-	-	-	-
SFH	24	80	6	20	-	-	-	-
TARA	29	88	4	12	-	-	-	-
**Age (years)**	-	-	-	-	5.77	3	0.124	-
20–29	45	96	2	4	-	-	-	-
30–39	38	84	7	16	-	-	-	-
40–49	15	88	2	12	-	-	-	-
50–59	8	73	3	27	-	-	-	-
**Sex**	-	-	-	-	-	-	-	0.700
Female	88	87	13	13	-	-	-	-
Male	18	95	1	5	-	-	-	-
**Relationship status**	-	-	-	-	2.26	4	0.688	-
In a relationship	36	86	6	14	-	-	-	-
Married	34	92	3	8	-	-	-	-
Single	33	89	4	11	-	-	-	-
Divorced	2	67	1	33	-	-	-	-
Widowed	1	100	0	0	-	-	-	-
**Number of dependents**	-	-	-	-	1.82	3	0.611	-
0	61	90	7	10	-	-	-	-
1–2	28	88	4	13	-	-	-	-
3–4	12	80	3	20	-	-	-	-
≥ 5	5	100	0	0	-	-	-	-
**Religion**	-	-	-	-	4.25	5	0.514	-
Areligious	5	71	2	29	-	-	-	-
Christian	77	88	11	13	-	-	-	-
Hindu	7	100	0	0	-	-	-	-
Jewish	2	100	0	0	-	-	-	-
Muslim	7	88	1	13	-	-	-	-
Other	8	100	0	0	-	-	-	-
**Years of work experience**	-	-	-	-	1.63	2	0.443	-
0–5	59	91	6	9	-	-	-	-
5–10	25	89	3	11	-	-	-	-
> 10	22	81	5	19	-	-	-	-
**Number of people in household**	-	-	-	-	0.44	2	0.801	-
1–5	96	89	12	11	-	-	-	-
> 5	10	83	2	17	-	-	-	-
**Type of housing**	-	-	-	-	-	-	-	0.221
Formal	105	89	13	11	-	-	-	-
Informal	1	50	1	50	-	-	-	-
**Access to healthcare**	-	-	-	-	-	-	-	1.000
Private	82	88	11	12	-	-	-	-
Public	24	89	3	11	-	-	-	-

PTSS, post-traumatic stress symptoms.

**TABLE 5:** The relationship between COVID-19-related factors and the occurrence of post-traumatic stress symptoms among mental healthcare workers from four Gauteng hospitals.

Variable	PTSS count (%)				Fisher’s *p*-value
	No		Yes		
	*n*	%	*n*	%	
**Participant care of COVID-19 patients**	-	-	-	-	1.000
No	28	90	3	10	-
Yes	78	88	11	12	-
**Someone close infected with COVID-19**	-	-	-	-	0.594
No	8	100	0	0	-
Yes	98	88	14	13	-
**Demise of someone close because of COVID-19**	-	-	-	-	0.572
No	57	90	6	10	-
Yes	49	86	8	14	-
**Infection of participant with COVID-19**	-	-	-	-	0.781
No	58	89	7	11	-
Yes	48	87	7	13	-
**Need for participant to self-isolate**	-	-	-	-	0.771
No	39	87	6	13	-
Yes	67	89	8	11	-

PTSS, post-traumatic stress symptoms; COVID-19, coronavirus disease 2019.

## Discussion

The prevalence of PTSS in this study was 11.7%. This is in keeping with a South African-based survey, including public psychiatric HCWs, in which the estimated prevalence rate of PTSD was 13%, and a cross-sectional survey conducted in China in which the estimated prevalence of PTSD was 13.7%.^31,32^ However, in relation to international research, this is lower than a comparison study carried out during the SARS outbreak and more recent studies performed in East Africa, Europe and East Asia in the context of COVID-19 in which the estimated prevalence of PTSD ranged from 25.7% to 80.5%.^22,33^ This discrepancy may result from the findings being influenced by different healthcare settings and various chronological periods during the COVID-19 pandemic in which these studies were conducted.^34^

In this study, the profession of the MHCW, particularly being a nurse, was a significant predictor of PTSS. This is consistent with a cross-sectional survey among nurses in the Free State province, South Africa, in which 44% of nurses were screened positive for PTSD.^35^ It is also in keeping with the findings of two cross-sectional studies based in China, which focused on the acute psychological effects of the COVID-19 outbreak on doctors and nurses working in affected hospitals.^36,37^ This finding is also in keeping with an international review of mental health disorders in nurses during the COVID-19 pandemic, which reported that nurses were at the highest risk of developing PTSD-like symptoms, highlighting the need for appropriate psychological support for this high-risk group.^38^ As was the case among participants in this study, the population found to be most at risk of developing PTSS included female nurses who worked in COVID-19-designated departments.^38,39^

Poor levels of perceived social support of MHCWs in this study were associated with a higher rate of PTSS than in those with good levels of perceived social support; however, the sample size was too small to make meaningful conclusions. Participants with greater BRCS scores reported less PTSS. This was in keeping with a cross-sectional survey of 863 medical workers in China, in which perceived social support and active coping strategies were negatively correlated to depression, anxiety and stress.^40^ In contrast, a South African-based survey revealed that moderate to high levels of coping behaviour and social support of hospital staff did not protect against psychiatric outcomes.^31^ However, these findings may have resulted from a smaller sample size in that particular study.

Global research found that being female, a younger HCW, earning a lower household income, working in a high-risk environment and undergoing quarantine were associated with a higher risk of PTSS.^8,13,25,26,27,28^ In addition, a study performed in Italy of 1379 HCWs found that having a colleague who died during the pandemic was associated with PTSS.^41^ However, none of the socio-demographic and COVID-19-related factors explored in this study were significant predictors of PTSS, which was out of keeping with findings in the literature.

There were limitations to this study. As a result of the cross-sectional nature, small sample size and specialised medical population, the findings may not be generalisable to other settings. It was also not possible to determine a causal relationship between pandemic-related exposure and associated outcomes. Data for this study were collected at a later stage of the pandemic, which may have impacted the degree of psychological distress experienced at that time. Participants were offered acute psychological containment but not referred for formal assessment in cases of probable PTSD. A strength of this study was that it addressed a significant research gap related specifically to the well-being of MHCWs.

## Conclusion

In this cross-sectional study, the prevalence of PTSS among MHCWs, as well as the risk and protective factors associated with this outcome, were studied. An elevated prevalence of PTSS was found and was significantly associated with the profession of the MHCW.

Based on these findings, it is recommended that existing employee wellness programmes be strengthened to promote mental well-being and improve resilience among MHCWs. It is recommended that regular screening programmes be implemented to identify staff members’ psychological distress and enable appropriate referrals. It is also recommended to provide additional resources to high-risk professionals, such as nursing staff, to regulate psychological well-being better. This could include departmental debriefing following a traumatic event, mental health education-based workshops targeting nursing staff and implementing resilience training programmes, as well as mindfulness exercises and relaxation techniques.
